# Preliminary CFD-Based Assessment of Additively Manufactured Muffler Insert Geometries

**DOI:** 10.3390/ma19122645

**Published:** 2026-06-19

**Authors:** Tomáš Zvoníček, Libor Novák, Petr Smolka

**Affiliations:** 1 Department of Physics and Materials Engineering, Faculty of Technology, Tomas Bata University in Zlín, Vavrečkova 5669, 760 01 Zlín, Czech Republic; t_zvonicek@utb.cz (T.Z.); lnovak@utb.cz (L.N.); 2 Centre of Polymer Systems, Tomas Bata University in Zlín, Třída Tomáš Bati 5678, 760 01 Zlín, Czech Republic

**Keywords:** muffler design, acoustic attenuation, turbulent kinetic energy, CFD simulation, 3D-printed inserts, flow-induced noise

## Abstract

This study investigates the impact of internal muffler geometry on flow-related dissipation characteristics potentially relevant to acoustic behavior using steady-state Computational Fluid Dynamics (CFD) simulations. Four variants were analyzed: an empty tube, considered to be a baseline model, a three-chamber baffle system, a single spiral channel, and a complex multi-channel insert manufacturable only via advanced additive technologies. Simulations were conducted in SimScale using a compressible flow model with the k-ω SST turbulence formulation. Key outputs included static pressure distribution and turbulent kinetic energy (TKE), both of which were evaluated as qualitative surrogate indicators associated with flow-induced energy dissipation phenomena. The results indicate that geometries incorporating spiral features modify flow redistribution patterns, pressure gradients and localized turbulence intensity, suggesting potential applicability for future acoustic optimization studies. The study highlights how additive manufacturing enables the integration of geometrically complex internal structures otherwise unattainable through conventional methods. By comparing pressure drop and TKE patterns with internal design features, the research offers a preliminary CFD-based framework for geometry screening and conceptual evaluation of muffler insert designs for automotive exhaust systems. This approach provides computational support for rapid comparative assessment prior to experimental validation and detailed acoustic analysis.

## 1. Introduction

In recent years, significant progress has been made in the use of complex internal structures and porous media for acoustic attenuation in confined systems such as silencers and mufflers. While experimental studies have traditionally focused on the measurement of sound absorption, reflectivity, or the mechanical-acoustic behavior of materials, increasing attention is now being paid to predictive modeling of acoustic performance using numerical methods. Among these, Computational Fluid Dynamics (CFD) has emerged as a robust tool for simulating compressible flows and sound propagation in intricate geometries, especially where direct experimentation is impractical or costly.

The internal configuration of silencers plays a critical role in determining acoustic efficiency. Studies such as Giedraitis and Kilikevicius have demonstrated how design modifications, particularly the use of multi-chambered configurations and smoothly varying cross-sections, can enhance acoustic attenuation by promoting energy dissipation [1]. Their findings are highly relevant to the implementation of 3D-printed inserts, which offer high design flexibility and can be tailored to specific flow and acoustic requirements.

Zeidan et al. explored the use of Tesla valves as passive flow control and damping elements. Their transient simulations showed that such geometries can effectively diffuse pressure spikes and redirect flow into vortical zones, thereby reducing the amplitude of pressure waves [2]. This confirms the potential of geometry-driven damping mechanisms, which can now be realized through additive manufacturing processes like SLM or DMLS.

Complementary research by Wurzinger et al. highlighted the importance of modeling not only acoustic pressure but also related properties such as impedance, wave speed, and resonance behavior in enclosed hydraulic systems prone to whistling or instability [3]. Their emphasis on time-domain resolution supports the idea that CFD-based acoustic analysis, especially under transient conditions, can provide deep insight into short-lived dynamic phenomena like shock waves or burst events.

Several investigations into automotive mufflers further reinforce the connection between internal baffle arrangement and overall system performance. Chandran demonstrated that the position and number of baffles significantly affect both sound suppression and pressure loss [4]. Rafique et al. examined extended chamber mufflers with micro-perforated panels and noted marked improvements in attenuation within the 500–2000 Hz range, without a proportional increase in backpressure [5]. Similarly, Liu et al. utilized time-dependent CFD to evaluate dissipative silencers under real exhaust flow and confirmed the predictive capability of such models in assessing both acoustic and aerodynamic performance [6]. There are also studies on enhancing the efficiency of solar thermal energy harvesting with modified baffler structures [7]. In the area of isolating low-frequency vibrations, different approaches are utilized [8].

These insights have been extended to 3D-printed muffler elements, including spiral chambers, periodic lattice structures, and multi-chambered designs. Simulation platforms like SimScale or COMSOL Multiphysics offer precise modeling of pressure fields, turbulence, and acoustic damping in real-world geometries. Notably, these tools allow comparative analysis of CNC-manufactured versus additively manufactured components, enabling developers to test more complex configurations without the need for costly prototypes [9].

Additive manufacturing further enables the fabrication of structures with features unattainable by conventional methods such as spiral baffles, gradient porosity, or internal periodic resonators. Technologies like SLS and DMLS facilitate the production of high-precision metal parts with defined acoustic properties [10].

From a physical perspective, sound attenuation in confined flow systems primarily occurs through two mechanisms: viscous dissipation, caused by velocity gradients near solid boundaries converting kinetic energy into heat, and thermal dissipation, resulting from cyclic compression and expansion of the fluid during acoustic wave propagation. These processes are especially relevant in muffler structures, where they contribute to broadband attenuation via local flow resistance and heat exchange. The Johnson-Champoux-Allard-Lafarge (JCAL) model provides a theoretical framework linking microstructural parameters, such as porosity, tortuosity, and characteristic lengths, to macroscopic acoustic performance and is widely used for modeling complex or additively manufactured porous media [11,12,13,14]. Structurally optimized materials with multiscale porosity extend the effective acoustic path length, enhancing both dissipation mechanisms. Periodic architectures like gyroid lattices allow frequency-selective damping and can be tuned through relative density, pore size, or wall thickness [15,16,17,18]. In spatially constrained environments such as automotive systems, these geometries enable acoustic tuning without altering macroscopic dimensions [19].

In the context of this study, a simplified yet physically meaningful approach has been adopted. CFD simulations based on steady-state compressible flow were used to investigate four muffler configurations: a baseline empty exhaust tube, a three-chamber system with baffles, a single spiral channel insert, and a complex multi-channel geometry only realizable through 3D printing. The pressure distribution and turbulent kinetic energy (TKE) across these variants were analyzed to assess their potential for sound attenuation. The aim is to evaluate how geometric manipulation enabled by additive manufacturing can influence acoustic behavior, with CFD serving as a non-invasive tool for performance prediction [20,21].

CFD simulations, including those run in environments such as SimScale or COMSOL Multiphysics, enable the detailed analysis of such mechanisms across both conventional and additively manufactured designs. While direct modeling of acoustic wave propagation requires transient analysis or specific acoustic solvers, the pressure drop and TKE in steady-state compressible flows provide valuable insight into how structural design influences sound attenuation [20,21,22].

In more advanced modeling approaches, the internal structure of muffler inserts, such as gyroid infill, lattice frameworks, or micro-perforations can be explicitly resolved. These methods, while computationally demanding, yield detailed maps of flow-structure interaction and pressure fields, allowing identification of so-called acoustically active zones [19]. However, for practical design evaluation and optimization, simplified models that use pressure and TKE as surrogate markers for attenuation efficiency are both feasible and informative. In contrast, transient simulations are required to capture phenomena such as acoustic resonance, wave propagation, or short-duration pressure pulses. However, these simulations demand fine temporal resolution, stable numerical schemes, and significantly higher computational resources, especially for large or geometrically intricate domains [21].

Similarly, the decision between compressible and incompressible flow modeling hinges on the Mach number of the flow and the presence of significant density changes. In applications like exhaust silencers, where high-pressure gases and compressible flow behavior dominate, it is critical to model density variations to capture expansion, compression, and shock-related behavior accurately [23]. In such systems, only compressible models can reliably simulate the interaction between flow structures and acoustic damping effects.

Contemporary studies in the automotive sector demonstrate that optimizing internal muffler geometry through the addition of baffles, perforated layers, or porous inserts can significantly improve acoustic attenuation without compromising engine backpressure or exhaust flow [6,24]. Additive manufacturing enables the realization of such complex geometries, making it possible to integrate spiral channels, Helmholtz cavities, and structurally graded porosity into compact muffler units [10].

By combining CFD-based flow analysis with the geometric flexibility of 3D printing, this research contributes to a deeper understanding of how internal structure affects pressure fields and turbulence, and how these in turn may influence flow-related dissipation potentially relevant to acoustic behavior in confined exhaust systems. The goal is to support the design of compact and manufacturable muffler concepts suitable for preliminary CFD-guided geometric evaluation using additive manufacturing techniques.

Although this study does not directly simulate acoustic wave propagation, it aligns with established approaches that use pressure gradients and turbulent kinetic energy as surrogate markers for flow-induced energy dissipation associated with acoustic attenuation mechanisms. These parameters provide physically meaningful indicators of energy dissipation via vortex shedding and flow instabilities—mechanisms commonly associated with sound damping behavior in confined flow systems. By focusing on steady-state compressible CFD analysis, the present work offers a computationally efficient yet insightful method for preliminary comparative evaluation of additively manufactured muffler geometries based on pressure redistribution and turbulence characteristics.

## 2. Materials and Methods

### 2.1. Simulation Environment and Approach

The simulations were conducted using the cloud-based platform SimScale (SimScale GmbH, Munich, Germany; cloud-based, available at https://www.simscale.com), applying a steady-state compressible flow model based on the Reynolds-Averaged Navier–Stokes (RANS) formulation, tensor form can be written as Equation (1) [25,26]. This approach was selected to evaluate geometry-induced pressure redistribution and turbulent kinetic energy (TKE) within the muffler domain. The aim was to identify flow regions associated with local turbulence generation, recirculation and pressure-gradient development, which may be relevant to subsequent acoustic interpretation.

(1) $$\frac{\partial (\rho U_{i})}{\partial t}+\frac{\partial (\rho U_{i}U_{j})}{\partial x_{j}}=\frac{\partial P}{\partial x_{i}}+\frac{\partial }{\partial x_{j}}\left[\mu \left(\frac{\partial U_{i}}{\partial x_{j}}+\frac{\partial U_{j}}{\partial x_{i}}\right)-\rho \bar{u_{i}^{\prime }u_{j}^{\prime }}\right]$$

where

*U* = mean flow velocity, *u*′ = velocity fluctuations due to turbulence, μ = molecular viscosity, and $-\rho \bar{u_{i}^{\prime }u_{j}^{\prime }}$ = Reynolds Stress term.

The Reynolds averaging process results in an additional stress term—the Reynolds Stress. To solve the RANS equations we need to express the Reynolds stress in terms of mean flow quantities.

The solution for the Reynolds stress term is given by the Eddy viscosity hypothesis/Boussinesq as [25,26]:

(2) $$-\rho \bar{u_{i}^{\prime }u_{j}^{\prime }}=\mu_{t}\left(\frac{\partial U_{i}}{\partial x_{j}}+\frac{\partial U_{j}}{\partial x_{i}}-\frac{2}{3}\frac{\partial U_{k}}{\partial x_{k}}\delta_{ij}\right)-\frac{2}{3}\rho k\delta_{ij}$$

where

$\mu_{t}$ = turbulent or eddy viscosity and $\delta_{ij}$ = Kronecker Delta

$$\delta_{ij}=\{\begin{array}{c} 1,\;if\;i=j\;\to Normal\;stress\;component\\ 0,\;if\;i\neq j\;\to Shear\;stress\;component\;\;\;\; \end{array}$$

Equation (2) is a combined equation for the shear and normal components of Reynolds stresses. Observing Equation (2) we realize that once we solve for the turbulent viscosity, we can solve the RANS Equation (1) [25,26].

Hence, the difference between different turbulent models is the methodology to calculate turbulent viscosity [25,26].

The steady-state formulation was adopted as a comparative early-stage design screening approach. This choice enables the evaluation of relative differences between internal geometries under identical operating conditions, particularly in terms of pressure loss, flow redistribution, and turbulence localization. However, the model does not resolve transient pressure-wave propagation, resonance effects, or frequency-dependent acoustic quantities such as transmission loss.

The turbulence model applied was the k-ω SST (Shear Stress Transport), known for its ability to accurately predict near-wall behavior and separation under adverse pressure gradients. Although this approach does not capture transient acoustic phenomena, pressure drop and TKE were evaluated as qualitative surrogate indicators of flow-induced energy dissipation mechanisms, rather than as direct measures of acoustic attenuation [20,21].

### 2.2. Geometry and Design Variants

**Figure 1:**
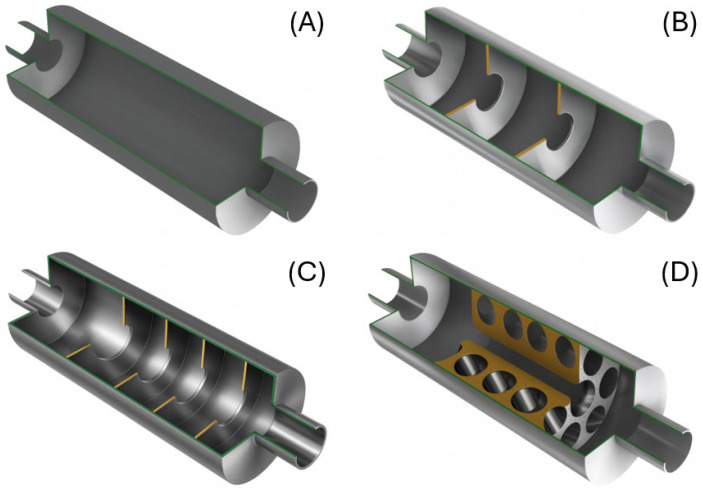
CAD models of for all four muffler configurations obtained from steady-state compressible CFD analysis. (**A**) Reference variant, (**B**) Three-chamber configuration with internal baffles, (**C**) Spiral insert, (**D**) Twisted multi-channel insert.

Four muffler configurations were analyzed:

Figure 1 presents the CAD models of the four muffler configurations evaluated in this study. These geometries were designed to isolate and compare the influence of internal flow-guiding structures on pressure distribution and turbulent kinetic energy (TKE) within the muffler body. Common muffler geometry details used for all CFD variants are listed in the Table 1.

(A) Baseline (smooth pipe, no insert):

The baseline model consists of a simple hollow cylindrical muffler with no internal structures or flow obstructions. It represents a straight-through exhaust design, providing an unimpeded flow path. This configuration serves as the control case for evaluating the impact of added geometrical complexity.

(B) Three-chamber baffle system:

This variant incorporates two internal baffles, dividing the muffler into three chambers. The baffles are intended to partially redirect the flow and disrupt its coherence, thereby increasing turbulence levels and promoting energy dissipation through simple geometrical intervention.

(C) Single spiral insert:

The third configuration introduces a spiral-shaped internal structure embedded within the cylindrical muffler. The spiral guides the airflow along a curved path, increasing the flow length and introducing rotational components to enhance turbulent mixing while preserving a centralized flow channel.

(D) Multi-channel 3D-printed insert:

The final geometry features a highly complex internal structure composed of six curved channels that twist around the central axis of the muffler. This design, enabled by additive manufacturing, extends the effective flow path by inducing a helical trajectory. The objective is to generate controlled turbulence and maximize acoustic damping through geometric intricacy.

All four variants used the same external muffler casing and inlet/outlet configuration. The only geometrical difference between the cases was the internal insert geometry; therefore, the common domain dimensions are summarized in Table 2.

### 2.3. Material Modeling, Boundary Conditions and Fluid Parameters

In all CFD simulations, the muffler walls and internal inserts were modeled as rigid, adiabatic, and non-deformable surfaces. No fluid–structure interaction or vibroacoustic coupling was included. The aim was to isolate the effects of internal geometry on pressure distribution and turbulent kinetic energy under identical wall and material assumptions, rather than to evaluate material-dependent acoustic or structural damping effects. All configurations were therefore assumed to be manufactured from the same sufficiently stiff engineering material, such as stainless steel or aluminum alloy.

This simplification allows the influence of geometry on the internal flow field to be evaluated independently of wall compliance, structural vibration, or material damping. While this approach neglects potential contributions of structural response to sound attenuation, it is suitable for early-stage comparative CFD screening, where the primary objective is to compare pressure redistribution and turbulence characteristics between alternative internal geometries.

As can be seen in Table 3, the inlet boundary condition was defined as a total pressure inlet of 0.14 MPa at 300 K, while the outlet was set to a static pressure outlet corresponding to atmospheric pressure of 0.101 MPa. This pressure difference was used as a simplified representative operating condition to generate pressure-driven compressible flow through the muffler domain and to enable direct comparison of all four geometries under identical loading. The same boundary conditions were applied to all configurations to ensure that observed differences in pressure drop and turbulent kinetic energy were caused primarily by internal geometry rather than by changes in operating conditions. Air was used as the working fluid, modeled as a compressible gas with ideal gas behavior.

### 2.4. Mesh and Solver Settings

Each geometry was discretized in SimScale using an automatically generated hexahedral-dominant finite volume mesh. Local refinement was applied in regions where elevated velocity gradients and flow separation were expected, particularly near narrow channels, baffle edges, curved internal walls, and inlet/outlet transitions. The same meshing strategy was applied to all four geometrical variants to maintain a consistent comparative basis.

The mesh resolution was selected based on numerical stability, residual convergence, and the consistency of pressure and turbulent kinetic energy fields. Since the present study is intended as a preliminary CFD-based geometry screening, rather than a fully validated high-fidelity numerical prediction, a complete mesh independence study was not performed. Nevertheless, the selected meshes provided stable convergence behavior and physically consistent pressure and turbulence distributions across the compared variants.

As can be seen in Table 4, the non-orthogonality and skewness remained within the platform-defined acceptable ranges and stable convergence was achieved; the mesh was considered suitable for preliminary comparative CFD analysis.

The flow field was solved using a steady-state compressible formulation of the Navier–Stokes equations combined with the k-ω SST turbulence model. Residual convergence was monitored for pressure, velocity components, and turbulence quantities. The convergence settings is displayed in the Table 5. The simulations were iterated until stable residual behavior was achieved and the monitored pressure drop and turbulent kinetic energy distributions no longer showed significant variation.

The numerical schemes were based on a steady-state formulation with cell-limited least-squares gradients, bounded upwind schemes for selected convective terms, Gauss linear limited Laplacian schemes, and linear interpolation. This setup was selected to support numerical stability in complex internal geometries with curved channels, baffle edges, and locally non-orthogonal cells.

### 2.5. Output Parameters

Primary simulation outputs included cross-sectional maps of static pressure distribution, cross-sectional maps of turbulent kinetic energy (TKE), and axial pressure profiles extracted along the muffler length for all four configurations. These outputs were selected to compare geometry-induced pressure redistribution, pressure-gradient development, and turbulence localization within the internal flow domain.

Pressure drop and TKE were treated as qualitative flow-related indicators associated with potential energy dissipation mechanisms. In the scope of the present preliminary CFD-based study, these quantities were not interpreted as direct acoustic performance metrics. Instead, they were used to support a comparative assessment of how different additively manufacturable internal geometries affect flow resistance, flow redistribution, and localized turbulence generation under identical boundary conditions.

### 2.6. Model Verification and Limitations

The numerical model was assessed through residual convergence, mesh quality diagnostics, and inspection of the resulting pressure and turbulent kinetic energy fields. Residual controls were set to an absolute tolerance of 1 × 10^−5^ for velocity, pressure, turbulent kinetic energy, and specific dissipation rate, and the simulations were evaluated based on stable residual behavior and physically consistent flow-field development. Mesh quality was checked using the diagnostics available in SimScale, including non-orthogonality, skewness, and cell-type distribution. The presented results should be interpreted as preliminary CFD-based comparative indicators of geometry-induced pressure redistribution and turbulence localization, rather than as fully validated quantitative predictions of acoustic performance.

## 3. Results

The following section presents the outcomes of the CFD simulations, focusing on the comparative effects of internal muffler geometry on static pressure distribution and turbulent kinetic energy (TKE) across the four design variants. These are evaluated as flow-related indicators of pressure redistribution and turbulence localization under identical boundary conditions.

### 3.1. Pressure Field Distribution

**Figure 2:**
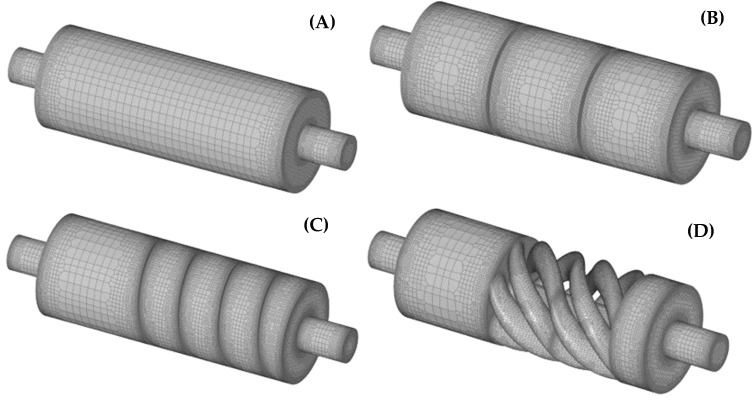
Computational mesh generated for all four muffler geometries used in the CFD. (**A**) Baseline variant, (**B**) Three-chamber system with internal baffles, (**C**) Single spiral insert, (**D**) Multi-channel 3D insert.

Figure 2 presents the distribution of static pressure within all four muffler variants obtained from the CFD analysis. The color scale indicates static pressure in kilopascals, with red regions representing high-pressure zones and blue corresponding to low-pressure areas near the outlet. The pressure fields are interpreted primarily as indicators of geometry-induced flow redistribution and pressure gradient development under identical boundary conditions.

(A) Baseline geometry:

The baseline configuration is a straight cylindrical muffler without internal structures and exhibits an almost linear pressure decrease along its length. The main pressure drop occurs directly before the outlet, where the gas expands into the surrounding volume. The internal pressure gradient is smooth and uniform, indicating limited flow obstruction and low resistance. This behavior is consistent with an unobscured flow path, which provides a reference case for assessing the influence of added internal geometrical features.

(B) Three-chamber baffle system:

In contrast to the reference model, the baffles introduce a stepwise pressure reduction across the three chambers. Each partition induces localized losses that slow the flow and redistribute static pressure, as evident from the more gradual color transition from red to green-blue hues. The resulting pressure dissipation supports the reduction in flow coherence and introduces localized pressure gradient regions. Such flow redistribution may be relevant to energy dissipation mechanisms.

(C) Single spiral insert:

The spiral geometry produces a controlled combination of resistance and guided flow. The pressure decreases smoothly along the helical path without sharp discontinuities or back-flow regions. The color gradient is evenly distributed, indicating a balanced pressure field and efficient flow stabilization. The outlet pressure approaches values slightly above atmospheric pressure (≈102 kPa), suggesting that the spiral structure promotes progressive pressure redistribution while maintaining flow continuity. This behavior may be considered favorable for further acoustic-oriented optimization.

(D) Multi-channel 3D-printed insert:

The most complex configuration, composed of six curved channels twisting around the central axis, achieves the most homogeneous pressure field among all variants. The inlet zone (left) shows a high-pressure region near 140 kPa, which gradually decreases along the length of the muffler. The color transition from red-orange to green-blue demonstrates a continuous and evenly distributed pressure drop without abrupt transitions. The mid-section reveals redistribution of the pressure field across multiple sub-channels, leading to enhanced spatial uniformity both axially and radially. At the outlet (right), the pressure approaches 100–102 kPa, close to atmospheric levels, without noticeable gradients or reflection zones. These results indicate that the additively manufacturable multi-channel design is effective in distributing the pressure gradient over a larger internal flow path. Among the investigated variants, this configuration shows the most favorable pressure redistribution characteristics for preliminary geometry screening.

### 3.2. Turbulent Kinetic Energy Distribution

**Figure 3:**
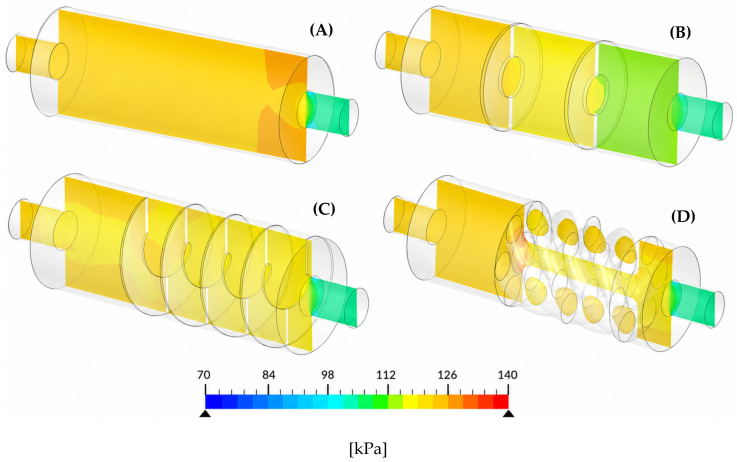
Static pressure distribution in kPa for all four muffler configurations obtained from steady-state compressible CFD analysis. (**A**) Baseline variant, (**B**) Three-chamber system with internal baffles, (**C**) Single spiral insert, (**D**) Multi-channel 3D insert. Note: Each image uses an individually scaled color bar to highlight internal pressure variations specific to the individual configuration.

Figure 3 shows the distribution of turbulent kinetic energy (TKE) within the four muffler configurations. The values, in units of m^2^/s^2^, are visualized on a logarithmic color scale, highlighting localized turbulence intensity throughout each geometry. In the present study, TKE is interpreted as a qualitative indicator of turbulence generation, flow separation and local energy redistribution.

(A) Baseline:

The TKE distribution in the baseline configuration is concentrated near the inlet and especially the outlet zones, where flow accelerates sharply and interacts with ambient conditions. The central volume of the muffler remains largely unaffected, with turbulence levels significantly lower across most of the domain. The maximum recorded value reaches approximately 1637 m^2^/s^2^. However, this peak is spatially localized and does not represent the overall turbulence level within the muffler body. The relatively low turbulence intensity in the central region is consistent with the unobscured flow path and confirms the suitability of this configuration as a reference case for evaluating the influence of internal geometrical features.

(B) Three-chamber baffle system:

Compared to the reference model, this configuration demonstrates clearly elevated turbulence levels within selected internal regions. Notable TKE intensities are observed between the internal baffles, particularly in the central region, with peak values approaching 1010 m^2^/s^2^. These localized zones of enhanced turbulence result from flow redirection and separation caused by the baffles. The results indicate that even a relatively simple baffle can substantially modify the internal flow structure and generate localized turbulence zones. From the perspective of preliminary geometry screening, this behavior suggests increased potential flow-induced energy redistribution compared with the unobscured baseline configuration.

(C) Spiral insert:

The TKE field in the spiral configuration reveals a more complex and spatially distributed turbulence pattern. The highest concentrations occur near the outlet and in the mid-sections of the spiral, where curvature and directional changes amplify vortex formation. Transitional zones between spiral turns exhibit moderate turbulence levels due to flow turning and shear interactions. In contrast, the core flow within the spiral channel remains relatively stable, with blue to green color zones indicating lower turbulence. The maximum TKE reaches around 773 m^2^/s^2^. This distribution suggests that the spiral insert provides a balanced flow behavior, combining controlled turbulence localization with relatively smooth flow guidance through the internal channel.

(D) Multi-channel 3D-printed insert:

The most geometrically complex design exhibits the lowest overall turbulence intensity among the four configurations. The TKE ranges from approximately 2 × 10^−8^ to 548 m^2^/s^2^, with most of the internal domain dominated by low-to-moderate values (blue and light blue zones). Higher turbulence appears only in the inlet and outlet transition regions, where the flow enters or exits the structured domain. Inside the central volume, the multiple curved channels promote gradual flow redistribution and reduce the formation of strongly localized TKE regions. This behavior suggests that the multi-channel geometry distributes the flow more uniformly across the available internal pathways. Among the investigated variants, this configuration therefore shows the most stable turbulence field and the lowest peak TKE, which may be advantageous for applications where controlled flow redistribution and reduced dynamic fluctuations are desirable.

### 3.3. Pressure Drop Along the Central Axis

**Figure 4:**
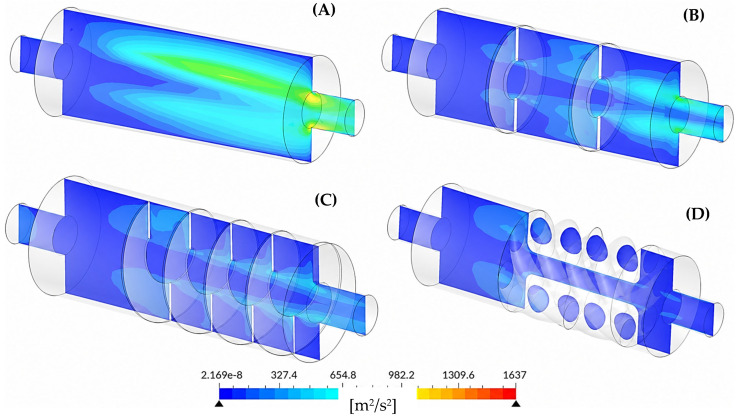
Turbulent kinetic energy in m^2^/s^2^ for all four muffler configurations obtained from steady-state compressible CFD analysis. (**A**) Baseline variant, (**B**) Three-chamber system with internal baffles, (**C**) Single spiral insert, (**D**) Multi-channel 3D insert. Note: Each image uses an individually scaled color bar to highlight internal TKE variations specific to the individual configuration.

Figure 4 presents the pressure distribution along the muffler length for all four design variants, as extracted from the CFD simulations. The pressure was evaluated at a central axis probe spanning the full axial length (0–375 mm). These profiles allow direct comparison of axial pressure decay, flow resistance and geometry-induced pressure redistribution across the different geometries.

(A) Baseline geometry:

The baseline configuration exhibits a gradual pressure decline from approximately 131 kPa at the inlet to ~102 kPa at the outlet. The central segment (~50–200 mm) shows a relatively flat pressure plateau, indicating limited internal flow obstruction and low pressure-gradient development. The sharp drop near the outlet reflects expansion into the ambient environment. This profile corresponds to a weakly modulated flow path with low hydraulic resistance, making it a suitable reference case for evaluating the effect of internal geometrical features.

(B) Three-chamber baffle system:

The baffle-based design exhibits a more nonlinear pressure trend. An initial slow decrease is followed by a steeper drop between 100 and 200 mm, associated with flow separation behind the internal partitions. This results in earlier and more distributed pressure loss. The peak inlet pressure reaches ~134 kPa, the highest of all cases, likely due to local compression near the baffles. This configuration demonstrates stronger flow modulation and pressure redistribution than the reference, indicating that even simple internal partitions can substantially alter the axial pressure profile.

(C) Single spiral insert:

The spiral-guided variant shows a smooth, continuous pressure decay from ~134.4 kPa to ~102 kPa. The curved geometry creates a gradual pressure gradient with no sudden drops or stagnation points. The mid-section curve inflection indicates progressive flow deceleration and distributed pressure redistribution along the spiral path. This geometry combines increased flow guidance with relatively smooth axial pressure decay, suggesting a balanced pressure response compared with the more segmented baffle-based configuration.

(D) Multi-channel 3D-printed insert:

The most complex design, featuring spatially twisted channels, achieves the smoothest and most uniform pressure gradient. The inlet pressure (~134.4 kPa) decreases steadily to ~102.8 kPa at the outlet, with no sharp transitions. This profile reflects distributed pressure-gradient development across the internal flow path, enabled by the additively manufacturable multi-channel domain. The curve suggests highly uniform pressure redistribution and stable flow guidance, with reduced abrupt pressure changes compared with the simpler internal configurations.

**Figure 5:**
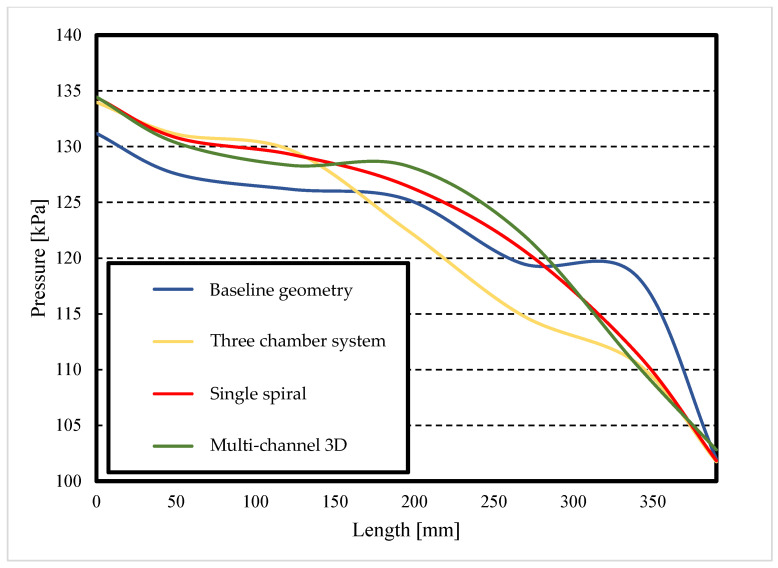
Pressure drop along the central axis of the muffler.

Overall, the comparison highlights a clear trend: as internal geometry complexity increases, the pressure profile becomes smoother and more evenly distributed, suggesting improved geometry-induced flow redistribution under the investigated operating condition, as shown in the Figure 5. While the outlet pressures converge across all designs, the rate and uniformity of pressure decline differentiate the configurations. The multi-channel 3D geometry demonstrates the most favorable axial pressure redistribution characteristics among the analyzed variants, combining smooth pressure decay with distributed resistance. These attributes are relevant for preliminary CFD-based screening of additively manufacturable muffler insert geometries, while direct acoustic performance must be evaluated in future studies using dedicated acoustic simulations or experimental validation.

## 4. Discussion

The results of the numerical simulations show that the internal geometry of the muffler substantially influences static pressure distribution, axial pressure decay, and turbulent kinetic energy (TKE) localization. Within the scope of this study, these quantities were evaluated as flow-related indicators of geometry-induced pressure redistribution and turbulence generation. The obtained results therefore provide a comparative basis for preliminary assessment of additively manufacturable muffler insert geometries.

The reference baseline configuration, featuring a smooth, unobstructed internal geometry, exhibited the least interference with the flow field. The pressure drop occurred almost linearly along the muffler length, with the main pressure gradient appearing just before the outlet. This behavior is consistent with a weakly modulated flow path and low hydraulic resistance. The central region of the muffler showed relatively low turbulence intensity compared with the inlet and outlet transition zones, confirming that the baseline geometry provides a suitable reference case for evaluating the influence of additional internal structures. However, because the flow is only weakly redistributed inside the muffler body, this configuration offers limited geometry-induced mechanisms for pressure-gradient redistribution or localized turbulence generation.

The baffle-based three-chamber configuration, comprising two partitions forming a three-chamber structure, introduced a more pronounced modification of the internal flow field. The presence of internal partitions introduced localized pressure drops, particularly within the central chamber. The simulation of turbulent kinetic energy revealed the formation of localized turbulence zones downstream of the baffles, which can be attributed to flow redirection, shear-layer development, and partial separation. These results suggest that even relatively simple internal partitions can substantially influence pressure redistribution and turbulence localization. From the perspective of early-stage geometry screening, the baffle-based design represents a manufacturable and structurally simple solution for modifying internal flow behavior.

A different flow response was observed in the single spiral configuration, enabled by additive manufacturing. The spiral channel forced the flow along an extended path, resulting in a smoother and more evenly distributed pressure drop. The pressure gradient was more gradual, indicating improved flow control. Turbulence visualizations showed that the spiral effectively generated localized turbulence near curved and transitional regions, while maintaining relative stability in the central domain. This combination of guided flow redirection and localized turbulence suggests that spiral geometries may be useful for controlled pressure redistribution in compact muffler inserts manufactured by additive technologies.

The multi-channel 3D-printed insert showed the most spatially distributed pressure field among the investigated variants. The network of organically shaped channels enabled uniform pressure distribution and gradual flow redistribution across the internal pathways. The pressure dropped smoothly across the entire structure, approaching atmospheric conditions at the outlet without abrupt changes. The TKE distribution showed that turbulence was concentrated mainly in inlet and outlet transition zones, while the central structured domain was characterized by low-to-moderate turbulence intensity. This suggests that the multiple curved channels promoted gradual flow redistribution and reduced the formation of strongly localized high-TKE regions. Among the analyzed configurations, this geometry provided the most favorable combination of smooth pressure decay, distributed pressure-gradient development, and stable turbulence behavior.

A comparative evaluation of all configurations indicates a clear trend: increasing geometrical complexity can substantially alter internal flow redistribution under identical boundary conditions. While the smooth reference design offered low-resistance flow, it provided limited internal pressure modulation. In contrast, baffle-based and spiral designs demonstrated that even basic geometrical modifications can significantly influence flow behavior. However, the 3D-printed domain variant exhibited the greatest potential, fully leveraging additive manufacturing capabilities for internal geometry optimization. The baseline geometry provided low-resistance flow but limited internal pressure modulation. The baffle-based configuration generated localized pressure losses and turbulence zones. The spiral insert produced a more gradual pressure decay through guided flow redirection. The multi-channel 3D-printed geometry provided the smoothest pressure redistribution and the most stable overall turbulence field among the evaluated variants. These findings demonstrate the potential of additive manufacturing to realize internal flow-guiding structures that would be difficult or impossible to produce using conventional manufacturing methods.

It is important to emphasize that the present study does not provide a direct prediction of acoustic performance. The steady-state compressible CFD model does not resolve transient pressure-wave propagation, frequency-dependent acoustic response, resonance phenomena, transmission loss, or vibroacoustic coupling. Therefore, pressure drop and TKE should be interpreted only as qualitative flow-related indicators associated with mechanisms that may be relevant to subsequent acoustic optimization.

The main contribution of this study is the demonstration of a preliminary CFD-guided workflow for comparing additively manufacturable internal geometries. Such an approach can support early-stage design screening by identifying how different internal structures influence pressure redistribution, flow resistance, and turbulence localization before more computationally demanding acoustic simulations or physical prototyping are performed.

Future work may extend the current CFD-based approach by incorporating equivalent fluid models, such as the Johnson–Champoux–Allard–Lafarge framework, to simulate the acoustic behavior of porous or complex structures with reduced computational cost. This would enable direct calculation of transmission loss and acoustic impedance in conjunction with structural optimization. Future work should also include additional operating conditions, transient simulations and experimental validation. For acoustic-specific evaluation, impedance-based methods or direct transmission loss simulations could be used to quantify the frequency-dependent acoustic response of complex or porous structures.

## Figures and Tables

**Table 1:** Common muffler geometry used for all CFD variants.

Parameter	Value	Unit	Note
Total muffler length	290	mm	Overall axial length of the computational geometry
Main chamber outer diameter	42	mm	External muffler body diameter
Main chamber inner diameter	38	mm	Fluid domain diameter inside the main chamber
Wall thickness	2	mm	Difference between outer and inner geometry
Inlet inner diameter	38	mm	Internal inlet pipe diameter
Outlet inner diameter	38	mm	Internal outlet pipe diameter
Inlet pipe length	52	mm	Included in the model
Outlet pipe length	52	mm	Included in the model
Fluid domain length used for axial probe	286	mm	Pressure extracted along central axis
Working domain	internal fluid volume air	—	Solid walls treated as rigid boundaries

**Table 2:** Insert-specific geometric features.

Variant	Insert Type	Number of Chambers/Channels	Main Flow Path Character	Additive Manufacturing Relevance
A	Baseline/empty tube	0	Straight unobstructed flow path	Reference geometry
B	Three-chamber baffle system	3 chambers/2 baffles	Segmented flow path with internal partitions	Manufacturable by conventional or additive methods
C	Single spiral insert	1 spiral path	Curved/helical guided flow path	Suitable for AM-enabled geometry
D	Multi-channel 3D insert	6 curved channels	Distributed multi-path flow	Complex geometry primarily enabled by AM

**Table 3:** Boundary conditions.

Boundary/ Domain Item	Type/Model	Value/Setting	Note
Inlet	Pressure inlet—total pressure	0.14 MPa	Applied to the inlet face
Inlet temperature	Fixed value	300 K	Prescribed at the inlet
Inlet turbulence	Automatic	SimScale default	Turbulence quantities automatically assigned
Outlet	Pressure outlet—static pressure	0.101 MPa	Atmospheric outlet pressure
Walls	No-slip wall	—	Applied to all solid boundaries
Wall thermal condition	Adiabatic	—	No heat transfer through the walls
Working fluid	Compressible air	Ideal gas	Density evaluated as pressure-dependent
Structural response	Neglected	Rigid walls	No fluid–structure interaction
Vibroacoustic coupling	Neglected	—	No acoustic wave propagation or structural-acoustic coupling

**Table 4:** Main mesh parameters and quality indicators for all analyzed geometrical variants.

Variant	Nodes	Volumes/Cells	Mesh Type	Non-Orthogonality Avg/99.99% [Range 0–88°]	Skewness Avg/99.99% [Range 0–10]	Status
A	175,265	149,784	Hex-dominant finite volume mesh	1.844/30.622	0.084/3.227	✔
B	557,823	458,068	Hex-dominant finite volume mesh	5.749/66.800	0.063/3.243	✔
C	8,224,538	6,595,889	Hex-dominant finite volume mesh	7.853/73.207	0.089/3.192	✔
D	4,204,531	3,304,130	Hex-dominant finite volume mesh	8.732/76.002	0.108/3.231	✔

**Table 5:** Solver and convergence settings.

Solver/Convergence Parameter	Setting/Value	Note
Analysis type	Steady-state compressible flow	Used for comparative geometry screening
Governing equations	Compressible Navier–Stokes/RANS	RANS (Reynolds-Averaged Navier–Stokes) formulation
Turbulence model	k-ω SST	k-ω SST (Shear Stress Transport) model
Working fluid	Air	Compressible ideal gas
Numerical method	Finite volume method	SimScale/OpenFOAM-based solver environment
Time treatment	Steady-state iterative solution	End time = 1000 s; Δt = 1 s used as pseudo-time iteration control
Write control	Time step	Write interval = 1000
Non-orthogonal correctors	1	Used to improve pressure correction on non-orthogonal meshes
Pressure reference cell	0	Default/reference pressure cell
Pressure reference value	1 × 10^5^ Pa	Reference pressure level
Velocity solver	PBiCGStab	Used for U velocity components
Pressure solver	GAMG	Geometric-algebraic multigrid pressure solver
Enthalpy solver	Smooth solver	Used for h
Internal energy solver	Smooth solver	Used for e
Turbulent kinetic energy solver	PBiCGStab	Used for k
Specific dissipation rate solver	PBiCGStab	Used for ω
Residual tolerance for velocity	1 × 10^−5^	Absolute tolerance
Residual tolerance for pressure	1 × 10^−5^	Absolute tolerance
Residual tolerance for k	1 × 10^−5^	Absolute tolerance
Residual tolerance for ω	1 × 10^−5^	Absolute tolerance
Maximum runtime	2 × 10^4^ s	Simulation control setting
Decomposition algorithm	Scotch	Used for parallel domain decomposition
Convergence assessment	Residual stabilization + stable pressure/TKE fields	Used to judge solution stability
Time differentiation	Steady-state	No transient acoustic propagation resolved
Gradient scheme	Cell-limited least squares	Limiter coefficient = 1
Default divergence scheme	Gauss linear	General divergence discretization
Velocity divergence scheme	Bounded Gauss upwind	div(phi,U)
Turbulent kinetic energy divergence	Gauss linear	div(phi,k)
Enthalpy divergence	Gauss upwind	div(phi,h)
Internal energy divergence	Bounded Gauss upwind	div(phi,e)
Specific dissipation rate divergence	Bounded Gauss upwind	div(phi,omega)
Laplacian scheme	Gauss linear limited	Limiter coefficient = 0.5
Interpolation scheme	Linear	Default interpolation

## Data Availability

The original contributions presented in this study are included in the article. Further inquiries can be directed to the corresponding author.
